# Proteomics analysis: inhibiting the expression of P62 protein by chloroquine combined with dacarbazine can reduce the malignant progression of uveal melanoma

**DOI:** 10.1186/s12885-022-09499-z

**Published:** 2022-04-14

**Authors:** Xifeng Fei, Xiangtong Xie, Ruwei Qin, Anqi Wang, Xuan Meng, Fei Sun, Yifan Zhao, Dongyi Jiang, Hanchun Chen, Qiang Huang, Xiaoyan Ji, Zhimin Wang

**Affiliations:** 1 Department of Neurosurgery, Suzhou Kowloon Hospital, Shanghai Jiaotong University School of Medicine, 118 Wanshen Street, Suzhou, China; 2 Department of Neurosurgery, Second Affiliated Hospital of Suzhou Medical College of Soochow University, Suzhou, China; 3 Department of ophthalmology, Second Affiliated Hospital of Suzhou Medical College of Soochow University, Suzhou, China

**Keywords:** Orthotopic and ectopic transplantation models of uveal melanoma, Transgenic EGFP nude mice, Differential proteomics and bioinformatics analysis, p62 protein, Chloroquine, Dacarbazine

## Abstract

**Background:**

Although uveal melanoma (UM) at the early stage is controllable to some extent, it inevitably ultimately leads to death due to its metastasis. At present, the difficulty is that there is no way to effectively tackle the metastasis. It is hypothesized that these will be treated by target molecules, but the recognized target molecule has not yet been found. In this study, the target molecule was explored through proteomics.

**Methods:**

Transgenic enhanced green fluorescent protein (EGFP) inbred nude mice, which spontaneously display a tumor microenvironment (TME), were used as model animal carriers. The UM cell line 92.1 was inoculated into the brain ventricle stimulating metastatic growth of UM, and a graft re-cultured Next, the UM cell line 92.1-A was obtained through monoclonal amplification, and a differential proteomics database, between 92.1 and ectopic 92.1-A, was established. Finally, bioinformatics methodologies were adopted to optimize key regulatory proteins, and in vivo and in vitro functional verification and targeted drug screening were performed.

**Results:**

Cells and tissues displaying green fluorescence in animal models were determined as TME characteristics provided by hosts. The data of various biological phenotypes detected proved that 92.1-A were more malignant than 92.1. Besides this malignancy, the key protein p62 (SQSTM1), selected from 5267 quantifiable differential proteomics databases, was a multifunctional autophagy linker protein, and its expression could be suppressed by chloroquine and dacarbazine. Inhibition of p62 could reduce the malignancy degree of 92.1-A.

**Conclusions:**

As the carriers of human UM orthotopic and ectopic xenotransplantation, transgenic EGFP inbred nude mice clearly display the characteristics of TME. In addition, the p62 protein optimized by the proteomics is the key protein that increases the malignancy of 92.1 cells, which therefore provides a basis for further exploration of target molecule therapy for refractory metastatic UM.

**Supplementary Information:**

The online version contains supplementary material available at 10.1186/s12885-022-09499-z.

## Background

Uveal melanoma (UM) metastasis results from the malignant progression of the disease, as well as the beginning of an adverse prognosis [1], which has a close association with the change of its molecular biological characteristics [2–5]. In relation to target molecule therapies, the selection of key target molecules has attracted the attention of many researchers. According to Gene Expression Omnibus (GEO) analysis results, Zhao DD [6] found that the metastasis and prognosis of UM was correlated with the PI3K/Akt signaling pathway. Liu R [7] maintained that as the key link in regulating a tumor's resistance to multiple drugs, it was a common pathway for many cancers, and its inhibitors were not effective for all cancers, such as UM. In the survival analysis using three independent groups conducted by Choi S [8], it was discovered that the NDUFB9, NDUFV2, CYC1 and CTNNB1 genes might be prognostic factors for UM, but no target molecule therapy research was conducted in that study. Moreover, Wang F [9] established a prognostic analysis web server (OSuvm) on the basis of data from TCGA and GEO databases, which can help guide researchers and clinicians to verify sensitive prognostic markers in UM in a quick and convenient manner, but it has not yet been empirically tested. In the current study, EGFP inbred nude mice, which spontaneously display a tumor microenvironment (TME), were used as model animal carriers. The UM cell line 92.1 was inoculated into the brain ventricle. The p62, a multifunctional autophagy linker protein, was selected from 5267 quantifiable differential proteomics databases of UM (92.1-A) undergoing ectopic growth in the murine brain and the original UM (92.1) cell line, as the core protein for causing the higher degree of malignancy observed in 92.1-A in comparison to 92.1. In addition, p62 protein expression was inhibited by chloroquine combined with dacarbazine, proving that inhibition of the p62 protein alleviated the malignancy degree of UM undergoing ectopic growth, which is valuable for further research regarding target molecule therapies for metastatic UM.

## Methods

### Cell culture

The 92.1 choroidal melanoma cell line was provided by the Eye and Ear Research Institute of the University of Pittsburgh Medical Center. 92.1 cell line was maintained in 37 °C, 5%CO2 incubator in RPMI1640 (Corning, USA) containing10% fetal bovine serum (FBS, Corning, USA).

### Animals

Four-to-six-week-old male and female EGFP transgenic nude mice at an average weight of 25 g were provided by our group [10]. All the animals were bred and maintained in the Specific Pathogen Free Animal Care Facility, Nasal1000 grade.

The animal protocol was in compliance with the Guide for the Care and Use of Laboratory Animals by the National Academy of Sciences and published by the US National Institutes of Health and the Principles of Laboratory Animal Care formulated by the National Society for Medical Research. The study was carried out in compliance with the ARRIVE guidelines. The protocol was approved by the Institutional Animal Care and Use Committee at Suzhou Kowloon Hospital, Shanghai Jiaotong University School of Medicine.

### Transplantation model of human uveal melanoma of brain ventricle in nude mice

Five nude mice were used, as previously described [11], after narcotizing the experimental mice, a mini-sized cranial drill was used to drill a hole at the position 0.22 mm posterior to the bregma and 1 mm left of the sagittal suture under the guidance of the stereotactic apparatus. Then, a microinjection needle was inserted into the hole at the depth of 2.5 mm, Fifteen microliters of cell suspension (2 × 105 cells) was injected under the control of a micropump in 10 min. The needle was then withdrawn after being retained in the hole for 5 min. 3 to 4 weeks later, 4 tumor-bearing mice were found. As required by the experiment, the tumor-bearing mice were sacrificed in due time, and the whole brains were taken out.

### H & E staining

Mice were sacrificed, then eyeballs, orbits, brain, heart, lung, and liver were also observed using the naked eye. Tissues were fixed with 10% paraformaldehyde, the specimens were embedded and sliced (5 μm). Staining was performed.

### Immunofluorescence labeling

As previous reported [12], tumor tissues were fixed with 4% phosphate-buffered paraformaldehyde, dehydrated with 20% sucrose solution, and cut into 30 μm coronal sections with a cryostat. All the sections were washed 3 times with PBS, 10 min each. The primary antibody HBM45 (1:100,Abcam UK) (diluted in 0.3% Triton X-100/PBS) was applied overnight at 4 °C. The sections were washed 3 times with PBS, 10 min each before the second antibody was applied. Sections were exposed to secondary antibody CY3(1:800,Beyotime China) at room temperature. Finally, sections were mounted with Vectashield medium (Vector laboratories) and coverslipped. Results were viewed using a confocal laser scanning microscope (Lecai TCS SP2, German).

### Monoclonal culture of 92.1-A cells

Fresh tumor tissues of one tumor-bearing mouse were gently aspirated using a pipette and placed in 6-well culture plates filled with 5 mL of 1640 culture medium. The culture medium was replaced every 3 days. After 2 weeks, the cells were transferred to a culture flask, and monoclonal subculture was repeatedly performed using the limiting dilution method [13].

### Cell cycle detection using flow cytometer

As we reported previously [14]. After trypsin digestion, cells in the logarithmic growth period was centrifuged at 1000 rpm for 5 min; the supernatant was discarded before adding the medium; after being measured by counting chamber, the solution was diluted into single cell suspension with 106 cells and then tiled in a 6-well plate. The plate was placed in the 5% CO2 incubator at 37 °C for 24 h of adherent growth. After incubation for 24 h, trypsin digestion and centrifugation, the cells were washed by PBS twice before addition of 70% ice ethanol and placement at 4 °C overnight. Fixed cells were centrifuged and washed by PBS again, followed by action by RNAaes at 37 °C for 1 h, addition of PI and staining at 4 °C for 1 h. At last, cell cycle was detected by flow cytometer.

### Detection of cell proliferation via CCK-8 assay

Both 92.1 and 92.1-A cells were cultured in RPMI1640 containing 10% fetal bovine serum and placed in a 5% CO2 incubator at 37 °C. The cells were washed 3 times with PBS and trypsinized to cell suspension. Cell suspension were inoculated to the 96-well plate (2000 cells/well) with 6 duplicate wells for each group; Every day, CCK-8 reagent (Dojindo Chemical Technology Co., Ltd) was added to each well protected from light, followed by culture in the incubator for 2 h and detection of OD value at 450 nm using the microplate reader. The cell survival rate was calculated. SPSS 22 software was adopted to analyze the OD value of two cell lines and GraphPad Prism 5 was used for plotting to demonstrate the differences between groups.

### In vitroinvasiveness test

Scratch assay as described in the following steps as we reported [15]. Briefly, horizontal lines spaced approximately 0.5–1.0 cm apart were evenly marked across the wells on the back of the 6well plates using a color pen. At least 5 lines passed through each well. Approximately 5 × 10 5 cells were injected into each well; The cells were incubated in serum containing solution and transferred to stem cell conditions 2 h later. The next day, a tip was positioned with a ruler to ensure, to every possible extent, that it was placed perpendicular to the horizontal lines drawn on the back of each plate. The cells were rinsed with PBS three times. The detached cells were discarded and the remaining cells were cultured in media containing serum. Samples were obtained and photographed 0, 24, and 48 h after incubation.

### Transwell assay

Transwell invasion assay were conducted with a Corning Inc. transwell chamber. Melted Matrigel was mixed with serum-free medium in a 1:8 rate. the upper compartment was precoated with 100 μl of Matrigel. After the glue is solidified, the remaining liquid in the plate was discarded, 200 μ l of warm serum-free medium was added, and retained at room temperature for 30 min after which the remaining culture medium was removed. 1 × 105 cells suspended in 200 μl serum-free medium were seeded in the upper compartment of the chamber and 500 μl RPMI1640 with 10% FBS were added to the lower compartment of the chamber. The cells were incubated for another 24 h. After that, the cells were fixed with 4% paraformaldehyde for 30 min and stained with 0.1% crystal violet. The non-migrating cells in the upper chamber were removed carefully using a cotton swab. The migrated cells were cells on the lower surface were photographed with microscope in five randomly selected visual fields and the migrated cells were quantified using SPSS.

### Key protein screening and protein interaction diagram

Protein chip making and differential proteins of 92.1-Aand 92.1 cells were completed by Hangzhou Jingjie Biotechnology Co., Ltd. The protein relationship was analyzed by STRING 10.5. Proteins were imported into string 10.55 protein database for analysis, and preliminary protein interaction diagrams were generated. The results mentioned above were imported into Cytoscape 3.6.1 for further protein screening Finally, proteins of which the up-regulation difference was less than 1.2-fold, and the down-regulation difference was more than 0.83-fold were deleted. The Gene Card gene library was used to query the function of the related protein which may induce 92.1 malignant progression.

### p62 knockdown of 92.1-A

**Fig. 1:**
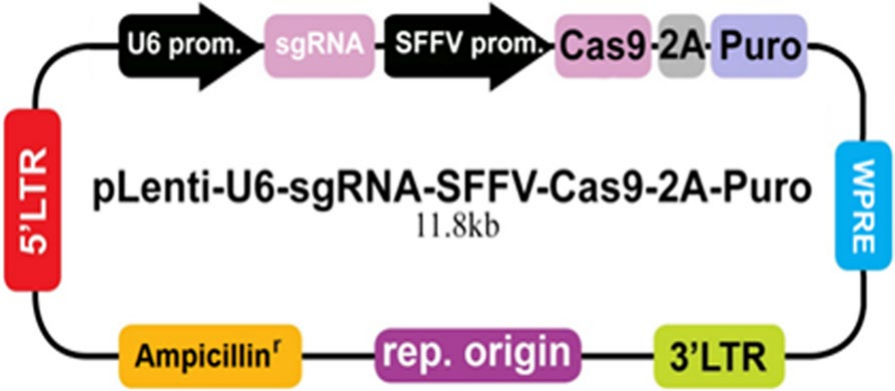
Plasmid diagram, Three sgRNA targets were designed, which were constructed into pLenti-U6-sgRNA-SFFV-Cas9-2A-Puro respectively

The cells in logarithmic growth phase were inoculated into 6-well plates. The cells were transfected by Lipofectamine 2000, when cells grown to about 60% confluence. The plasmid (Fig. 1 Applied Biological Materials abm, Canada) was mixed with Lipofectamine 2000 at room temperature for 15 min. Mixture was added to the cells culture medium and cultured for 6 h after which medium was changed to complete medium. After 24 h, the plasmid can be used in the following experiments.

### Over expression of p62 in 92.1 cells

92.1 cells were placed in a 6-well plate. After 24 h, when cells grown to 40%- 60% confluence, the cells were infected with lentiviral overexpressed of p62 (Applied Biological Materials abm, Canada). 5 ng / μ l Polybrene was added to improve the efficiency of virus infection.6 h later, cell culture medium was changed completely, and the transfection efficiency was observed under fluorescence microscope. After 24–48 h, the cells were collected for protein extraction.

### Western blot

As previously described [14], cells from each group were collected and placed in a 1.5 mL EP tube, followed by addition of appropriate amount of lysate for completely lysis and ultrasonic testing in the ice bath sing ultrasonic processor; after centrifugation at 2000 rpm for 15 min at 4 °C, the supernatant was extracted and stored at − 20 °C for further use. The oncentration of proteins extracted above was quantified sing BCA Protein Quantification Kit; 5 × loading buffer as added to the sample, followed by boiling at 100 °C or 5 min to denature the proteins. After processing by DS-PAGE, the proteins were transferred to the NC embrane using protein transfer device and then locked by 5% skimmed milk powder for 1 h. After addition of p62 antibodies (Abcam UK) and incubation vernight at 4 °C, the proteins were washed by BST three times before 1 h of fluorescent secondary ntibody incubation and another three times of TBST ashing; Western Blot imaging software Odyssey nalyzer was used to detect and produce images.

### Human uveal melanoma subcutaneous transplantation model in nude mice

Both 92.1 and 92.1-A cells in logarithmic growth phase were inoculated to 36 nude mice totally at the concentration of 1 × 106 cells/animal. After two weeks, when the tumor grew to the extent that vernier caliper could be used for measuring, the animals were divided into A) control group; B) chloroquine (CQ) treatment group; C) dacarbazine (DTIC) treatment group; and D) CQ + DTIC treatment group. Both drugs were intraperitoneally injected. The dosage of chloroquine was 50 mg / kg, intraperitoneal injection every day; dacarbazine was 50 mg / kg, o intraperitoneal injection every three days. The drug treatment lasted for three weeks. The body weight and tumor size of modeled mice were measured every 3 days. At the end of the experiment, as previously described, mice were anesthetized and sacrificed. The tumor tissue was removed and weighed in the aseptic condition. Paraffin section and immunohistochemical staining were conducted to analyze the traditional pathological and molecular pathological features of xenografts.

### Immunohistochemical staining

Immunohistochemical staining was performed for the paraffin sections of the transplanted tumor tissue according to the antibody instructions. The steps were as follows in briefly: 1) add in goat serum to block heterogenetic antigens after PBS hydration and endogenous peroxidase blocking with 0.3% hydrogen peroxide; 2) add in the detection p62 antibody (1: 500, Cell Signaling Technology); 3) incubate them overnight at 4 °C; 4) add in the corresponding biotin-labeled second antibodies for 1 h (Vectastain elite ABC kit) and followed by three 5-min washes with PBS. The sections were incubated with Vectastain elite ABC reagent for 30 min and followed by three 5-minwashes with PBS; and 5) seal the sections with neutral resins after DAB color development and hematoxylin counterstaining.

### GEPIA survival analysis

The GEPIA database (http://gepia.cancer-pku.cn/) was used to conduct survival analyses.The browser searched http://gepia.cancer-pku.cn/index.html, selected the Survival Plots, and typed SQSTM1 in the data box below the Gene. Then we selected Overall Survival in the “Methods”. Next selected Quartile as the Group Cutoff value, that is, those higher than 75% are high expression Group, those lower than 25% are low expression Group; Selected Yes for HR and 95% confidence interval, and monthly in “Axis Units”; Selected Uveal melanoma (UVM) in “Datasets”, Then click “Add” and click Plot to generate the survival graph of SQSTM1.

### Statistical analysis

The experiment was duplicated at least 3 times. Graph-Pad Prism 5 software was used for imaging analysis; one-way ANOVA and Student’s t-test were employed for data statistical processing, where the data were represented by mean ± standard deviation (X ± SD). P < 0.05 was defined as statistical significance.

## Results

### 1-A was more malignant than 92.1

**Fig. 2:**
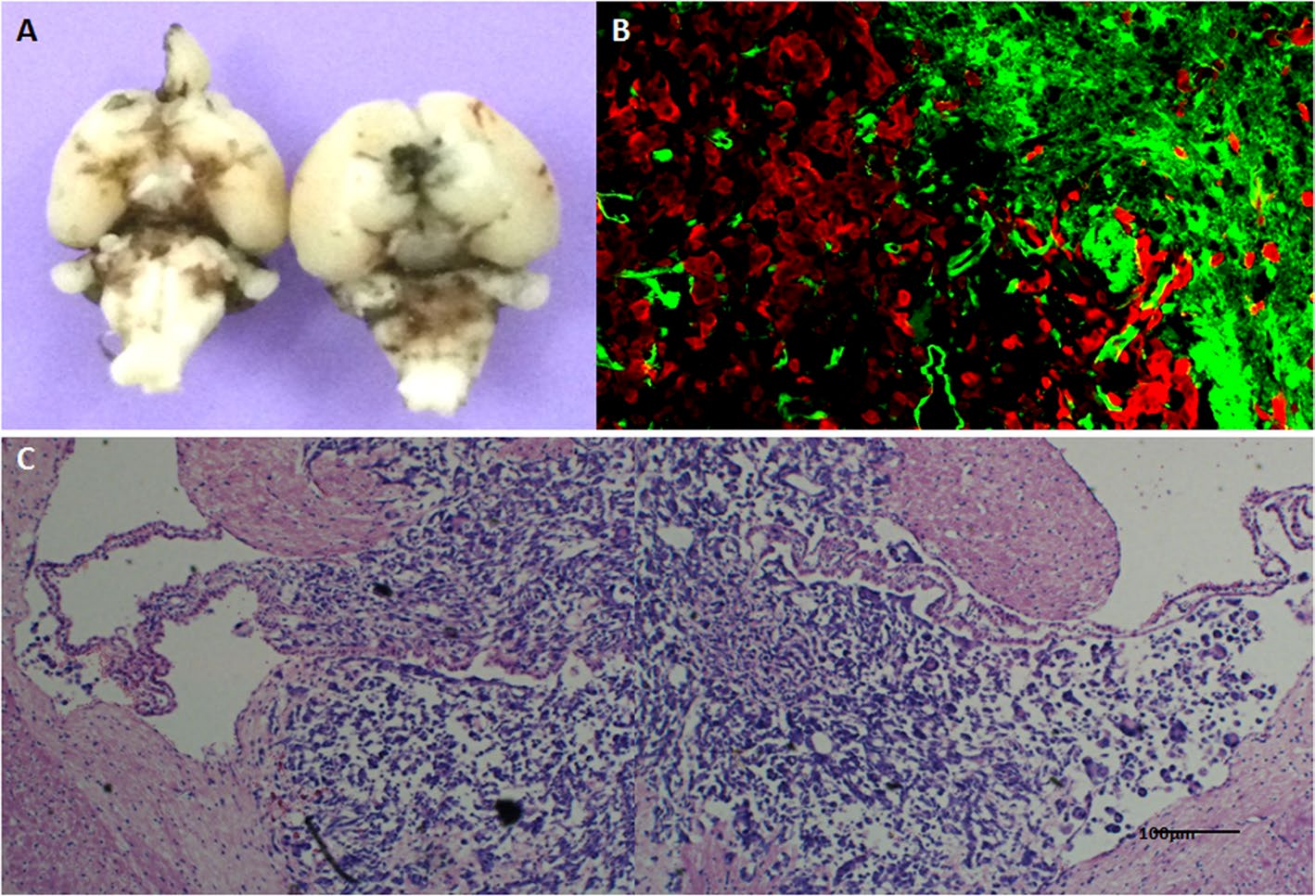
92.1-A transplantation tumor of brain ventricle in EGFP nude mice (scale 100 µm). **A** Black UM cells spread widely into the dorsalis/ventral cistern and sulci. **B** Fluorescent tracing showed that red UM cells (HBM45 positive cells) spread widely to the nearby and distant green host tissues, while there were sporadic green host choroid plexus tissues in the tumor mass. **C** HE staining showed tumors mass of UM cells distributed symmetrically in both hemispheres in a butterfly shape

**Fig. 3:**
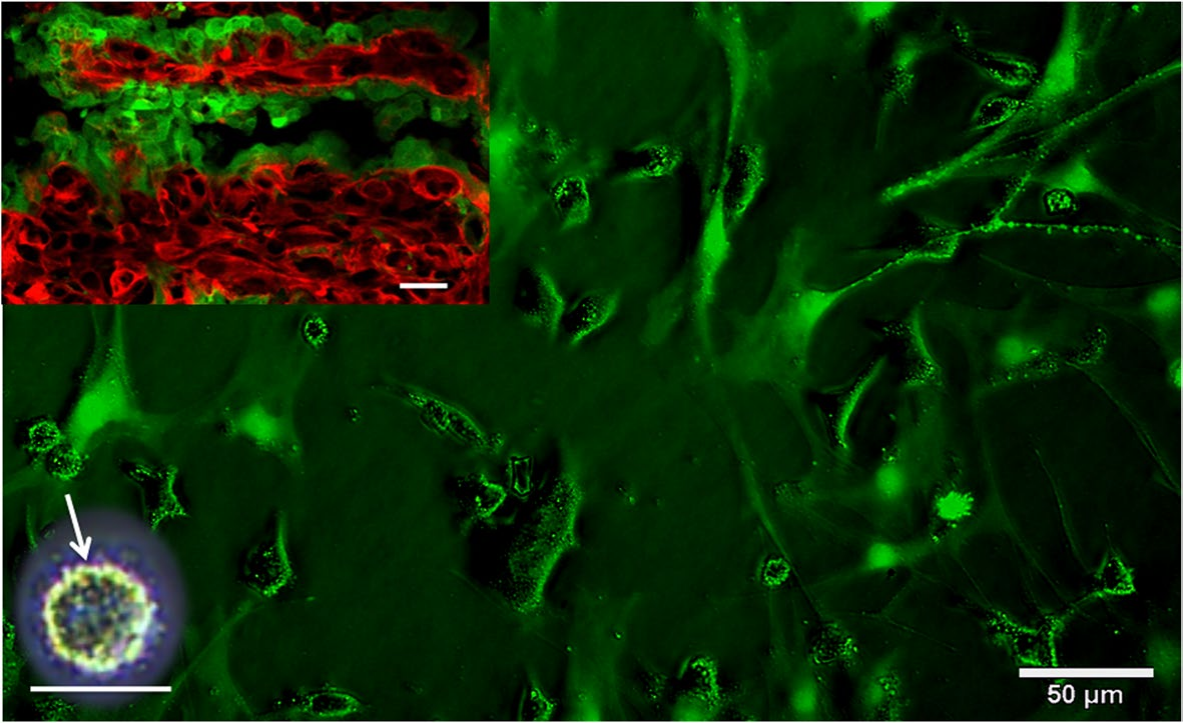
Primary culture of ectopic xenografts of 92.1(scale 50 µm). The red 92.1 cells (HBM45 positive cells) were colonized and expanded on the green choroidal plexus (upper left). Under the phase contrast microscope, the green cells with pseudopodia extension were the host cells, and the cells with many granules and dark color were 92.1 cells, among which the small round cells with abundant granules were spheroids under the white light microscope (lower left)

**Fig. 4:**
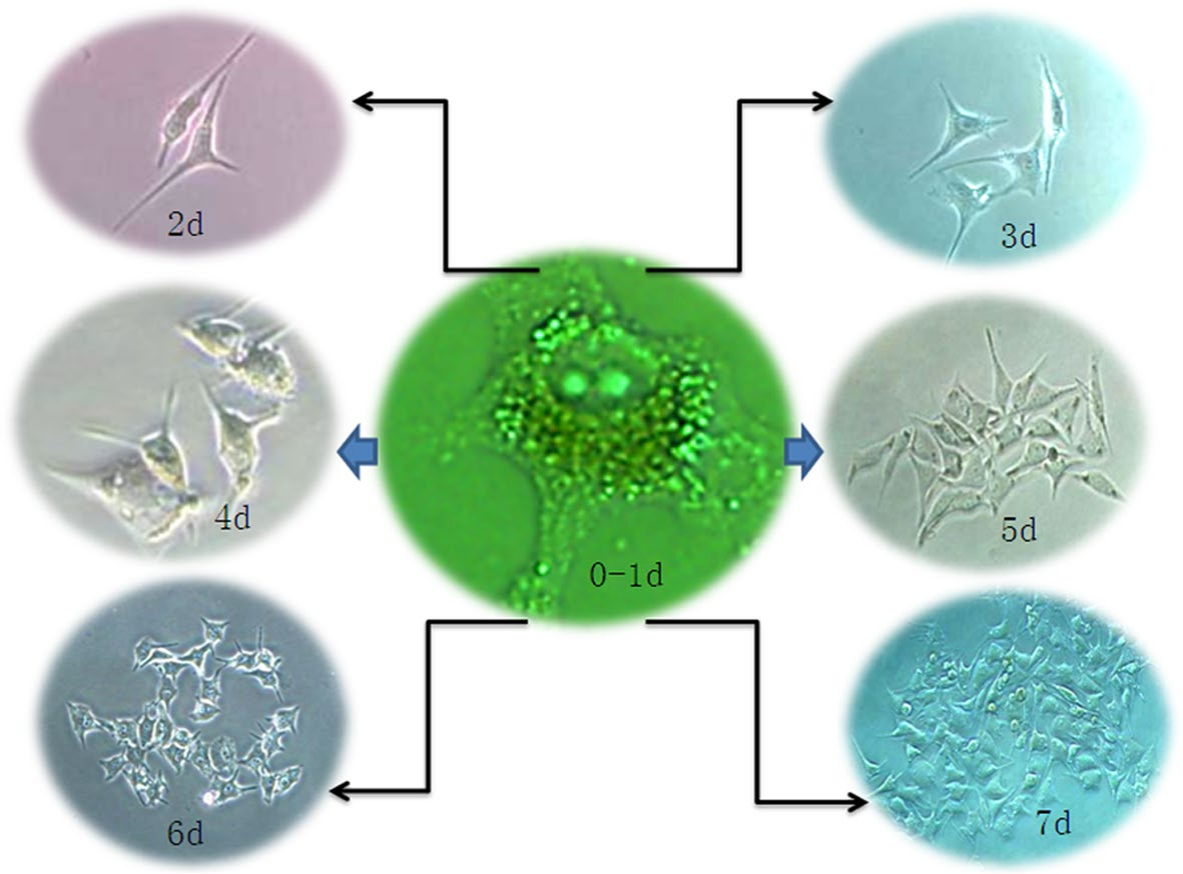
Monoclonal culture of 92.1-A cells from transplanted tumor tissue. 92.1-A cells were cultured by the limited dilution method. The cells divided once every 24 h from 2 to 4d, and the division was accelerated obviously from 5 to 7 d., After 7 days, the cells grew to 80% density and needed to be passaged

**Fig. 5:**
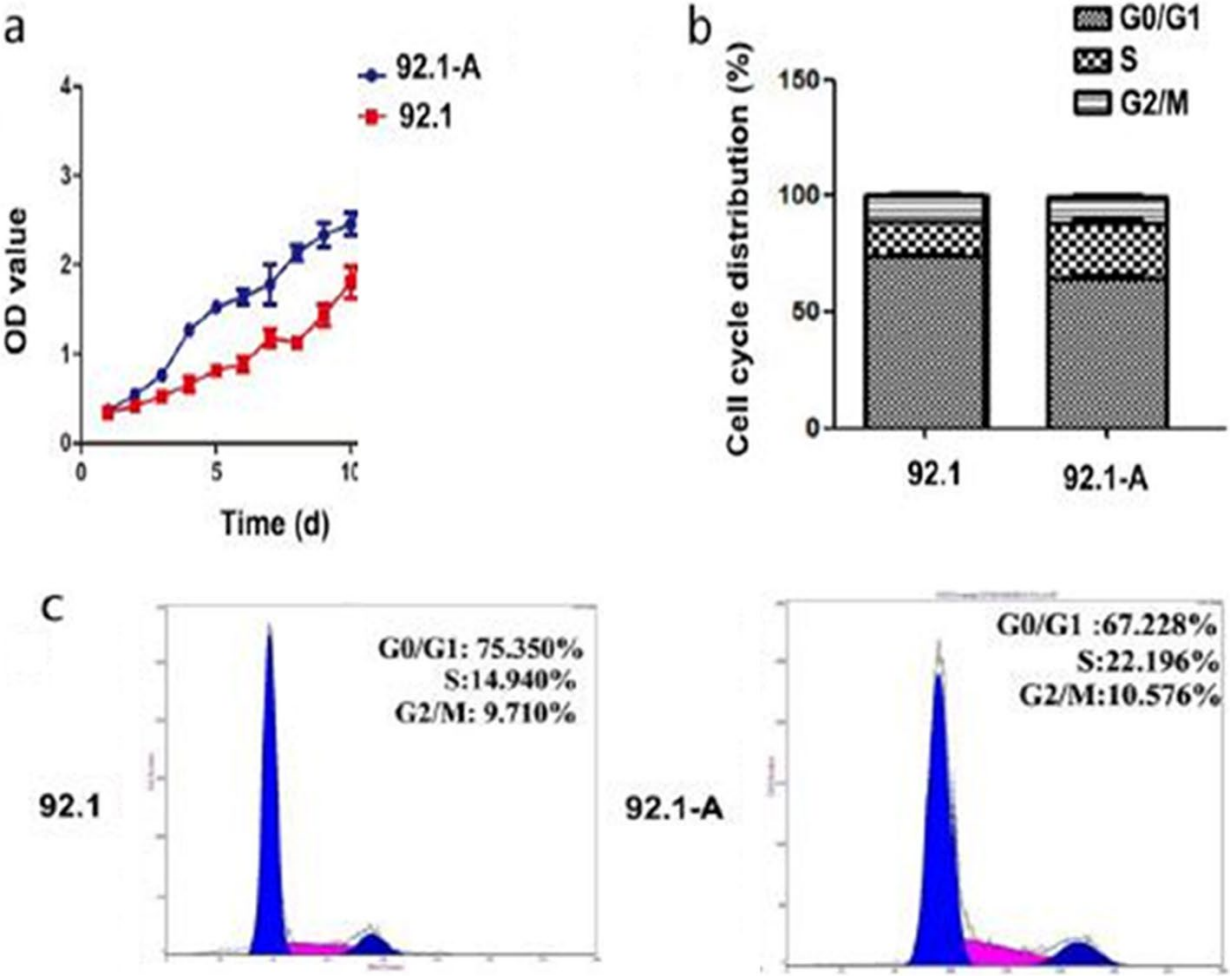
Cell proliferation and cell cycle: The cell proliferation curve indicated that, from the fourth day, the proliferation rate of 92.1-A was significantly faster than that of 92.1 (**a**). According to the data of cell cycle detection (**c**),the percentage of S phase and G2-M phase cells in 92.1-A was significantly higher than that in 92.1(*P* < 0.05), which indicated that 92.1-a cell division was vigorous (**b**)

**Fig. 6:**
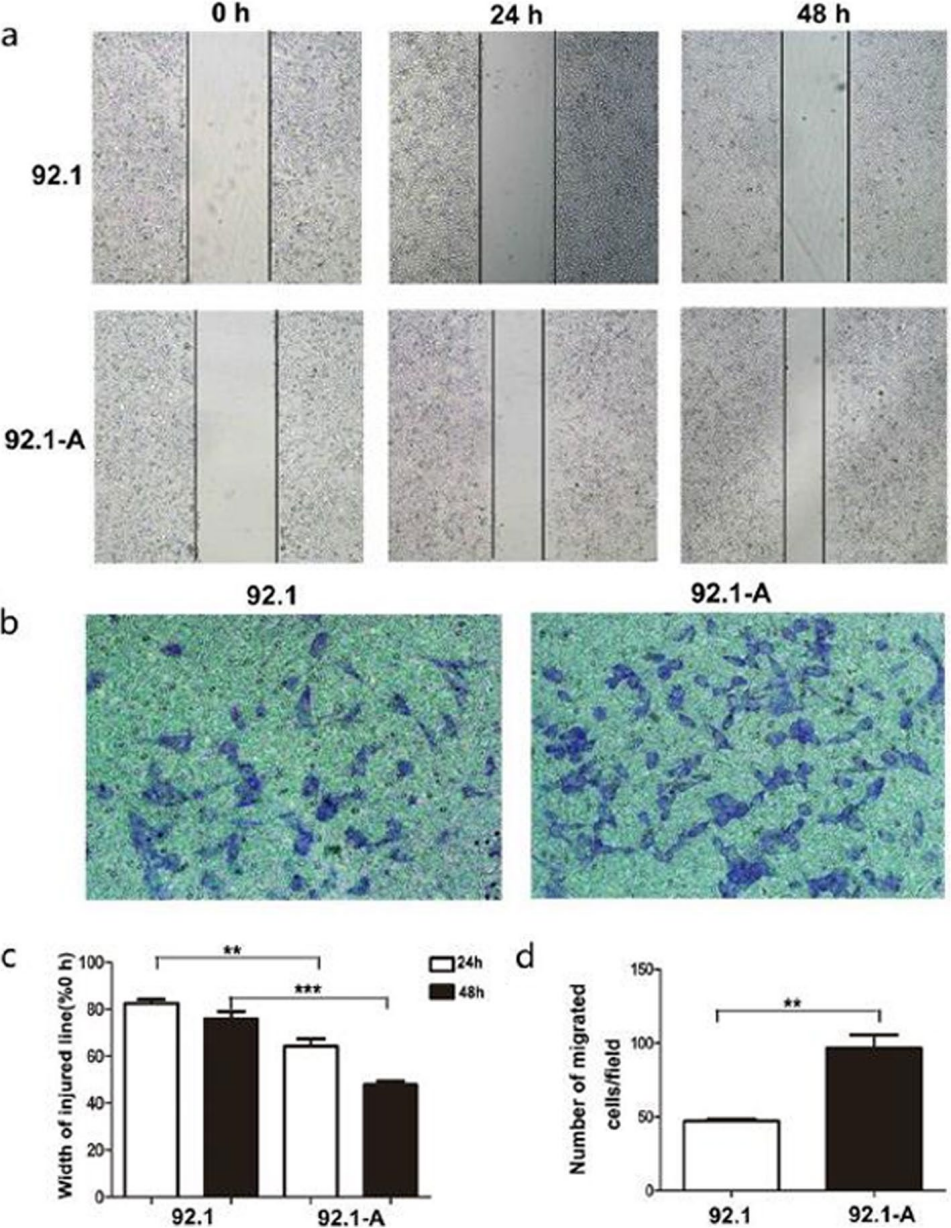
Invasiveness tests of cancer cells in vitro. Scratch assay (**a**) and Transwell test (**b**) showed that 92.1-A had stronger migration and invasion ability. **c** and **d** The statistical graphs of A and B respectively, ** *P* < 0.01,*** *P* < 0.001

The 92.1 UM cell line was derived from the human orbit and established by Waard-Siebinga ID et al. [16] in 1995. In order to study whether the biological characteristics of ectopic (ventricular) growth (simulated metastasis) were more malignant, the 92.1 cell suspension was inoculated into the brain ventricle of EGFP transgenic nude mice[17], and the solid tumor model of intraventricular metastasis was successfully established (Fig. 2). The results revealed that when 92.1 was inoculated into the brain, it spread widely into the subarachnoid space. Moreover cancer cells infiltrated in the choroidal plexus and along the white matter, forming a butterfly-like tumor, similar to highly malignant glioblastoma multiforme, and spread further as showed by fluorescence-traced cancer cells. These results indicated that transplanted tumor of ectopic (ventricular) growth was highly malignant. Furthermore, the ventricular metastatic tumors were cultured in vitro, and the tumor cell line 92.1-A was obtained after strict monoclonal re-culturing (Figs. 3 and 4). In addition to providing pure and comparable cell samples for subsequent proteome detection, CCK8 was additionally employed to compare the in vitro amplification abilities of the 92.1 and 92.1-A cells. It was found that 92.1-A had a significantly stronger amplification ability than 92.1 (Fig. 5). Moreover, the invasion abilities of the two cell lines in vitro were compared using a wound healing assay and a Transwell assay (Fig. 6), which showed that the invasion abilities of 92.1-A were remarkably stronger than that of 92.1.

### Databases of 92.1-A and 92.1 differentially expressed proteins were established based on proteomics

Hangzhou PTM Biotechnology Co., Ltd. was entrusted to detect the quality of monoclonal cells 92.1-A and 92.1, and the differential expression protein databases were established. In brief, the proteins were extracted, trypsinized, labeled by Tandem Mass Tags (TMT), classified by high performance liquid chromatography and analyzed via liquid chromatography-mass spectrometry tandem, followed by analysis via database searches and bioinformatics analysis. There were 6081 identifiable proteins obtained, of which 5267 were quantifiable. With 1.2 times given as the change threshold and *p* < 0.05 following *t*-test as the standard requirements, 335 proteins were up-regulated and 302 proteins were down-regulated in the 92.1-A compared to 92.1 group, among all quantified proteins. Subsequently, the functions of differentially expressed proteins were classified using commonly applied bioinformatics software GO and KEGG [18–20], and GO enrichment and KEGG pathway analysis were carried out.

### The key protein increased the malignant progression of 92.1-A

**Fig. 7:**
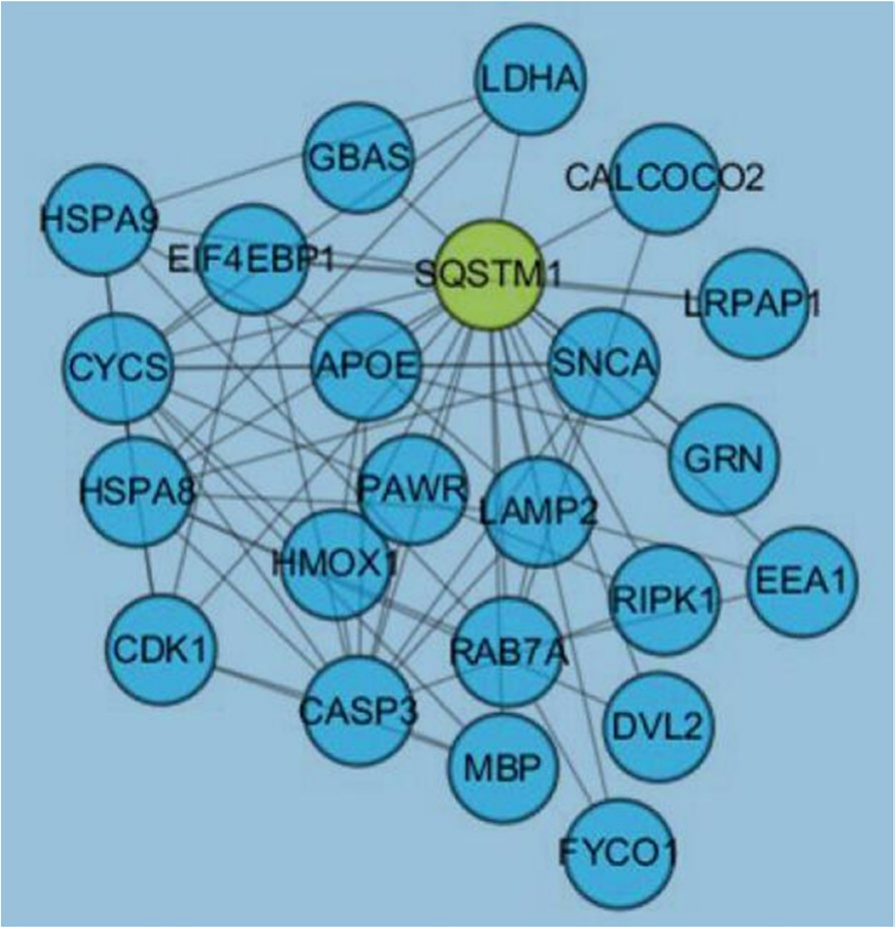
Visual contact diagram of the target protein and the other 22 proteins in the UM inflammatory microenvironment. According to the connection line, P62(SQSTM1) has the most extensive relationship with other proteins

Although the above-mentioned 637 differentially expressed proteins were obtained via statistical processing, and all of them had certain biological significance(s), most of the changes may occur with the molecular event (i.e., the malignant progression) of 92.1, therefore there was a need to select and highlight the responsible proteins. Hence, these data were initially imported into the STRING 10.55 protein database in order to generate an interaction network. Using Cytoscape3.6.1 for further screening, among the 23 proteins finally selected, Sequestosome 1 (SQSTM1, p62) was one of the most widely associated proteins with the other 22 proteins in the network diagram (Fig. 7), therefore it was tentatively designated as the key protein causing the higher malignancy degree observed in 92.1-A when compared to 92.1. This was further verified below.

### The regulatory abilities of key proteins were verifiedin vitro

**Fig. 8:**
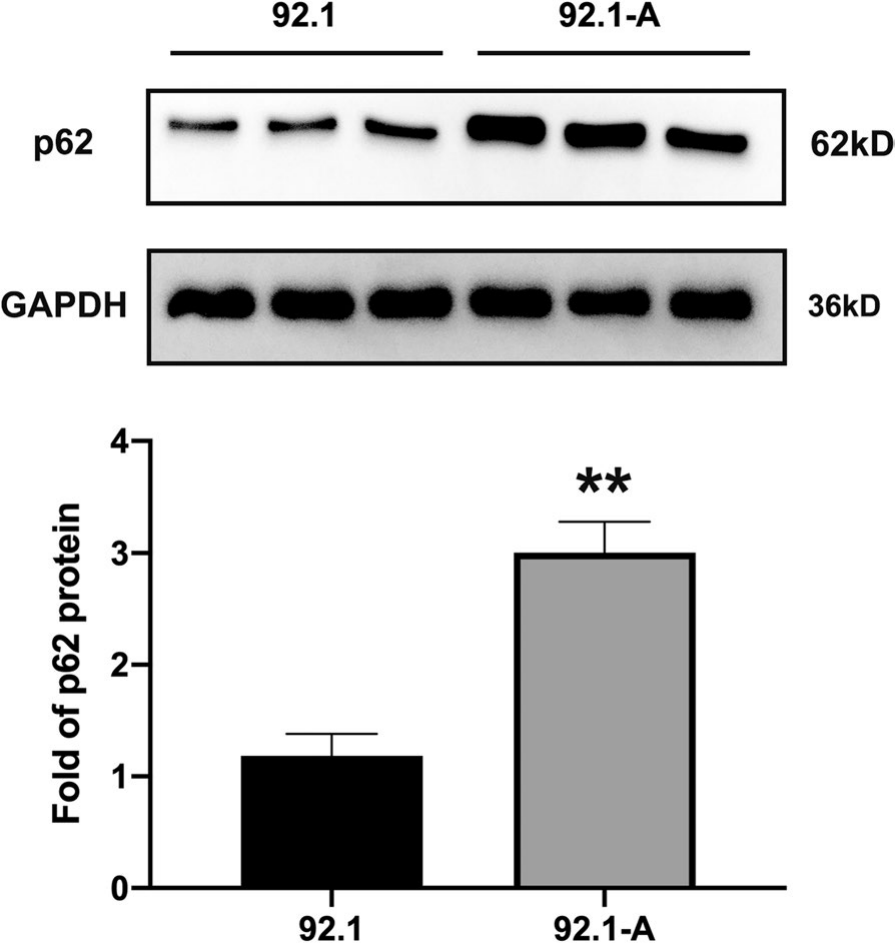
Expression of p62. **A** The expression of p62 in 92.1-A cells was significantly higher than that in 92.1 cells; **B** Statistical result of A. * *P* < 0.05

**Fig. 9:**
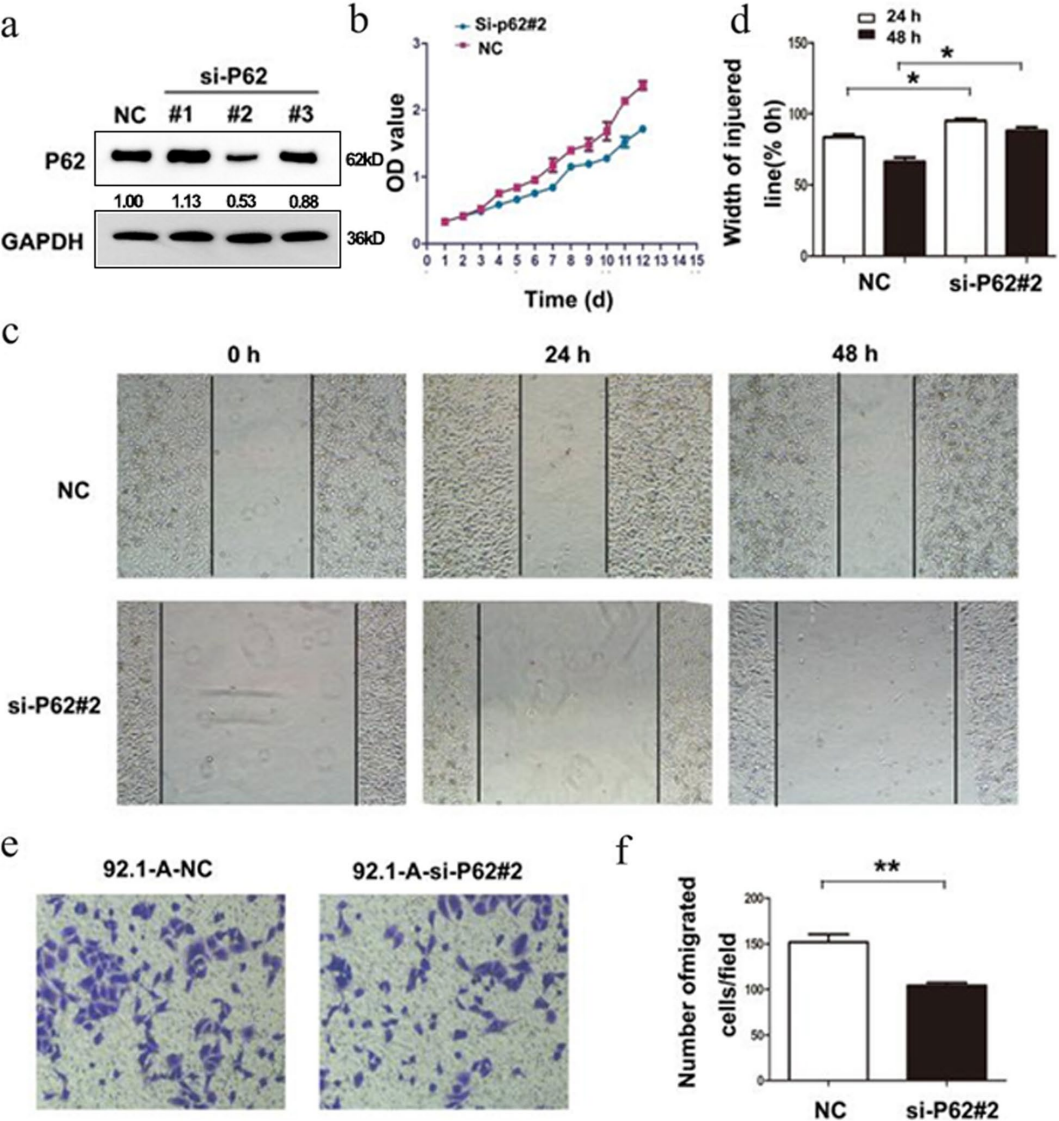
Cell biological changes after p62 expression knockdown (**a**): Among the three plasmids with different targets, the expression of p62 decreased most obviously Interfered by si-P62#2. **b** Compared to the control group, p62 knockdown can significantly reduce the proliferation rate of 92.1-A; **c** Scratch assay showed p62 knockdown can reduce the migration ability of 92.1-A cells, statistically significant; **d** Statistical result of (**c**). * *P* < 0.05; **e** Transwell assay showed p62 knockdown can reduce the invasion ability of 92.1-A cells, statistically significant; **f** Statistical result of (**e**). * *P* < 0.01

**Fig. 10:**
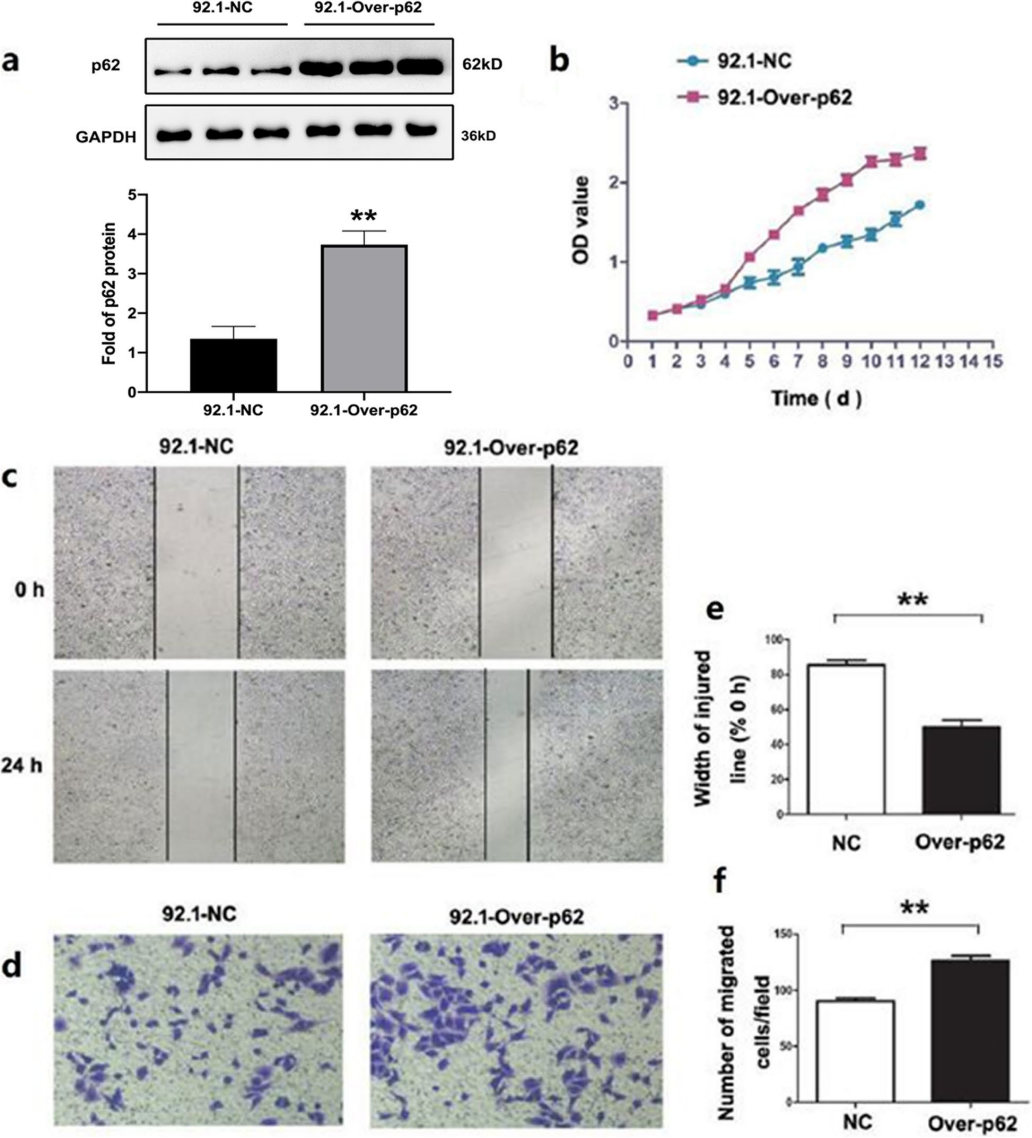
Cell biological changes after overexpression of p62. **a** p62 expression was increased after p62 overexpression in 92.1 cells; **b** Overexpression of p62 enhanced the proliferation ability of 92.1 cells; **c** Overexpression of p62 enhanced the migration ability of 92.1 cells; **e** Statistical result of (**c**). ** *P* < 0.01; **d** Overexpression of p62 enhanced the invasion ability of 92.1 cells; **f** Statistical result of (**d**). * *P* < 0.05

Firstly, western blotting detection results indicated that the p62 protein expression in 92.1-A was higher than that in 92.1 (*p* = 0.023), which was consistent with the protein array results (Fig. 8). The expression of p62 was knocked down in 92.1-A and it was discovered that the cell proliferation, wound healing and invasion abilities of 92.1-A were all decreased due to the decline in p62 protein expression (Fig. 9). On the contrary, after 92.1 cells had been infected with lentivirus overexpressing p62, the increased expression of p62 protein enhanced the cell proliferation, wound healing and invasion abilities (Fig. 10).

### Chloroquine and dacarbazine inhibited p62 protein expression in transplanted tumors

**Fig. 11:**
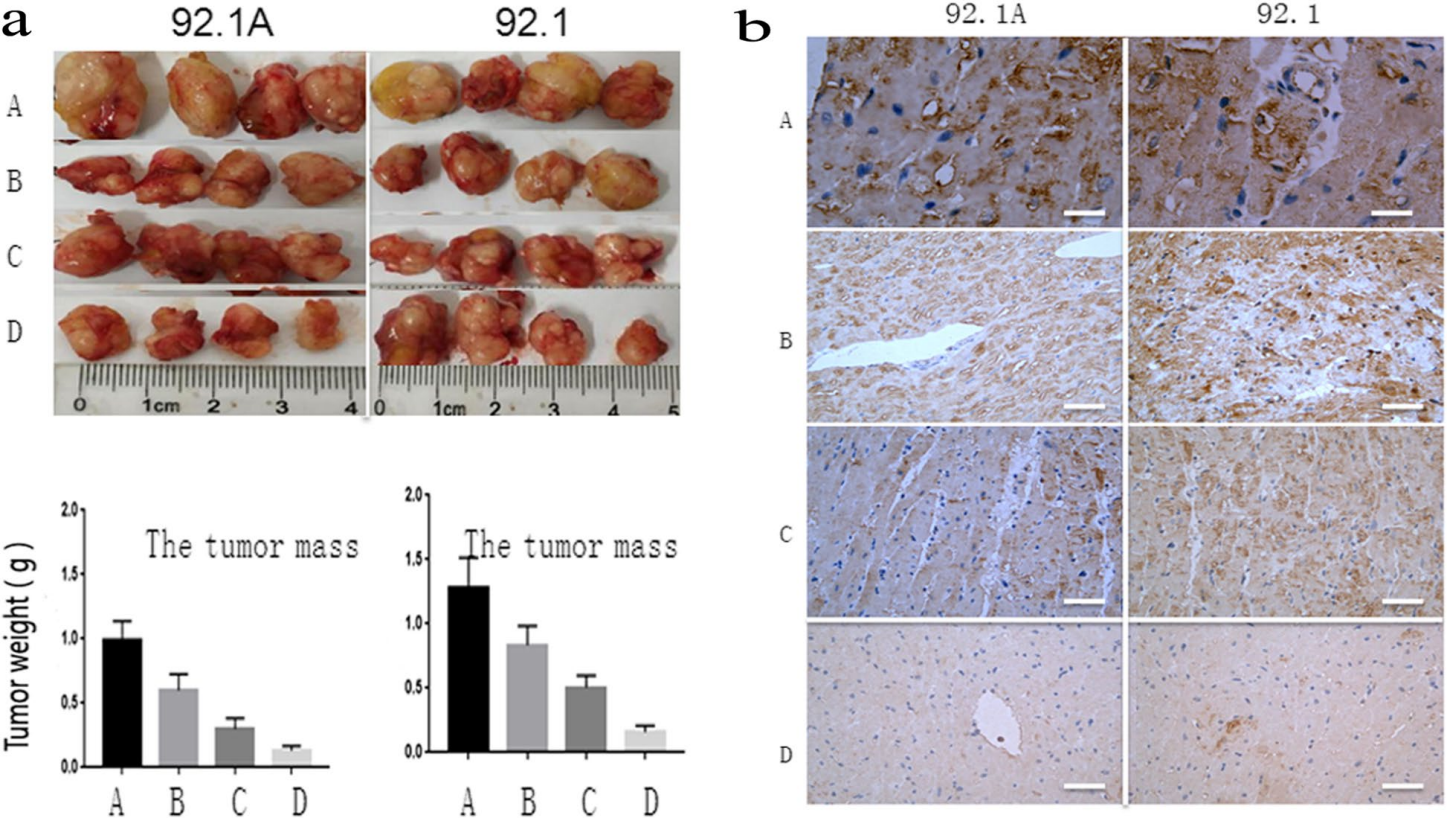
Chloroquine and dacarbazine inhibited tumor growing by inhibition of p62 expression (**a**) For the end of the experiment,Transplanted tumor photo graph and Transplanted tumor weight Statistical graph; **b** the expression of p62 protein in different group. (A was control Group, B was group of chloroquine, C was group of dacarbazine and D was group of chloroquine + dacarbazine)

During in vivo experiments, the p62 inhibitor chloroquine was used to control the proliferation of transplanted tumors, and the expression levels of the p62 protein were observed, using dacarbazine, a traditional medicine for treating melanoma, as a control. With the blank control (Group A, *n* = 4), chloroquine (Group B, *n* = 4), dacarbazine (Group C, *n* = 4) and chloroquine + dacarbazine group (Group D, *n* = 4), the statistical analysis of tumor masses and p62 protein expression showed that: (1) 92.1-A showed lower tumor masses than 92.1, which may be as a result of the higher expression levels of p62 in the 92.1-A cells. (2) Compared with Group A (blank control), treatment in Group C (dacarbazine) inhibited tumor amplification, which is consistent with the clinical findings. Moreover, the effect in Group D (chloroquine + dacarbazine) was better than those observed in any of the other groups, however, the exact mechanism needs to be further analyzed (Fig. 11).

### Significance of p62

**Fig. 12:**
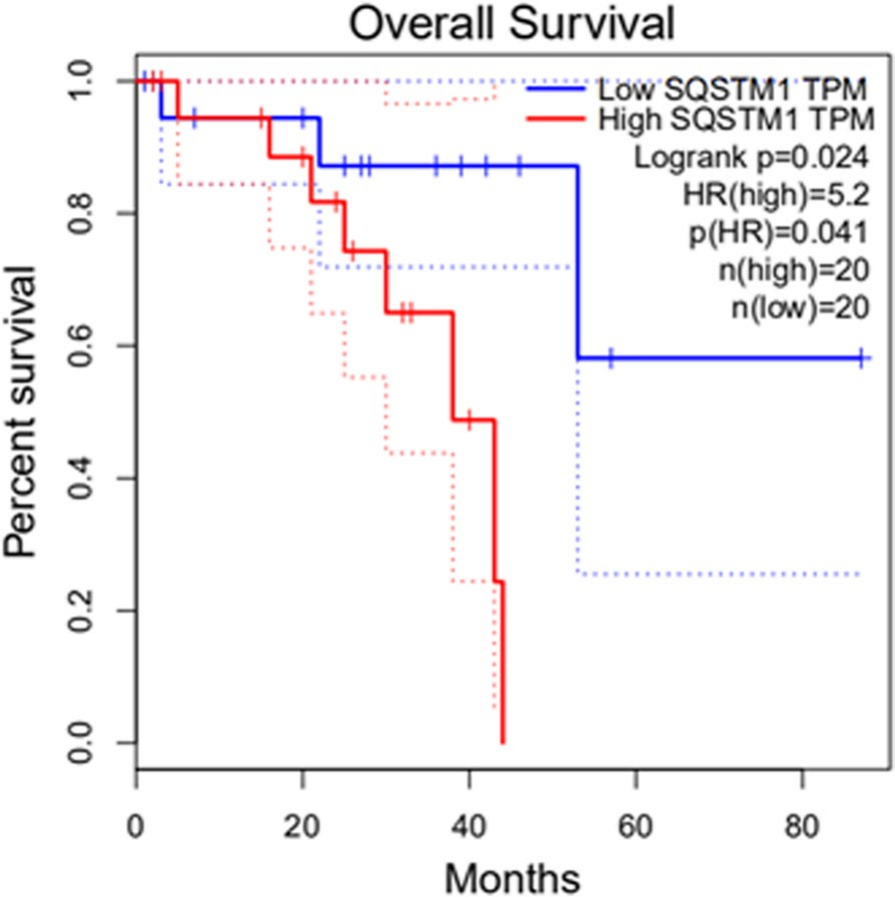
The relationship between P62 and UM prognosis. Result from GEPIA database indicated that patients with high p62 expression have a shorter survival time. * *P* < 0.05

SQSTM1 was input into the GEPIA database [21], and then the whole survival, quartile, and UVM were selected successively. The results highlighted that high expression of SQSTM1 was a high-risk factor for the lowest survival rate of UM patients (Fig. 12).

## Discussion

Patient with uveal melanoma is inevitably ultimately to death, due to its metastasis, mainly to the liver. In relation to the treatment of UM metastasis, Mariani P [22], in 2012, advocated to resect metastatic foci, which was put forward on the premise of a lack of effective target molecule therapies, and in fact it is still a palliative therapy. The 2015 UK UM guidelines [23] and the 2020 Irish and German UM guidelines [24] contained no target therapies, and there are no FDA-approved UM target molecule therapeutic drugs in the USA, indicating that this research has not officially been carried out clinically anywhere in the world. The reason for this is that although UM is clinically and biologically different from cutaneous melanoma (CM), most of the treatment options for advanced stage UM can refer to CM. In comparison, UM has a much lower efficacy rate and a much higher treatment-related toxicity than CM [25, 26]. Additionally, there are many high-quality guidelines for CM nursing, which promote clinical and diagnostic norms in a standardized manner [27, 28]. Despites this, there are few UM research articles and a lack of large-scale randomized controlled trials, which makes it difficult to prove the clinical effectiveness of interventions and create a solid foundation for evidence-based treatment decision-making. Another obstacle is that nursing of UM patients is highly interdisciplinary, involves ophthalmology, tumor, interventional radiotherapy and transferred organs, all of which require high-level guidelines suitable for multidisciplinary management, but presently no guidelines are available [29].

In the context of above-mentioned difficulties, although BRAF and mitogen-activated protein kinase (MEK) inhibitors have proved to contribute remarkably to the survival of CM patients, as per the meta-analysis by Theresa S [30], MEK inhibitors exerted no effect on UM. Fortunately, Truong A [31] reported that chloroquine was sensitive to MEK1/2 inhibition of GNAQ/11 mutant CM, which was also effective for the murine model of metastatic UM. They confirmed that the target molecule was GNAQ/11 mutant, which led to cancer cell death by inhibiting autophagy or lysosomal function. Although it was p62, and not the GNAQ/11 mutant, that was reported in this study, the two should be consistent as both of them involved autophagy or lysosome actions. From another point of view, the key target molecule for regulating UM metastasis may be found through high-throughput bioinformatics analysis. Crabb JW [32] established the differential expression databases of three clinical specimens of UM primary and metastatic foci with proteomics five years ago. Among the 28 proteins that were significantly upregulated and 30 proteins that were significantly downregulated, the fatty acid binding protein (FABP3) increased most remarkably. Unfortunately, the protein p62 reported upon in the present study was not included in these down or upregulated proteins.

P62 is a multifunctional protein produced by stress and acts on autophagy, it is also related to the occurrence and development of diverse diseases. Reports related to the target molecules of cancer metastasis include P62 is a New Tumor Target for Osteolytic Metastasis [33], Overexpression of P62 Can Induce Autophagy by Promoting ERK Signaling Pathway to Promote Proliferation, Migration and Invasion of Nasopharyngeal Carcinoma Cells [34], P62 Promotes Prostate Cancer Metastasis by Maintaining HDAC6 Level, Inhibiting Autophagy Flux and Promoting Epithelial-Mesenchymal Transition [35], P62 Can Promote the Growth and Metastasis of Skin Tumors and Can be Induced by Ultraviolet Radiation [36], and P62 Promotes CM Progression by Inhibiting the Decay of A Group of Selective Transfer-Promoting Factor mRNA [37]. As such, p62, as a new tumor target for the malignant progression of UM, can be effectively suppressed by combination treatment using chloroquine and dacarbazine, which has not been reported on before. However, the data reported in this study only comes from the database of differential protein expressions between 92.1 and 92.1-A which are not fully representative enough. As it was pointed out by Jager MJ [38], it is necessary to strive from the current diagnosis and treatment to achieve the final goal, that is, to develop a treatment method that can protect eyesight, effectively treat metastasis, and ultimately save lives.

## Conclusions

As the carriers of human UM orthotopic and ectopic xenotransplantation, transgenic EGFP inbred nude mice clearly display the characteristics of TME. The brain TME increased the malignant progression of tumor cells. In addition, the p62 protein optimized by the proteomics is the key protein that increases the malignancy of 92.1 cells, and the p62 inhibitor chloroquine could inhibit the proliferation of transplanted tumors, which therefore provides a basis for further exploration of target molecule therapy for refractory metastatic UM.

## Supplementary Information

**Additional file 1: Supplementary Figure 1.** Expression of p62. The expression of p62 in 92.1-A cells was significantly higher than that in 92.1 cells. The original images of the blots in Figure 8. **Supplementary Figure 2**. Among the three plasmids with different targets, the expression of p62 decreased most obviously Interfered by si-P62#2. The original images of the blots in Figure 9a. **Supplementary Figure 3.** p62 expression was increased after p62 overexpression in 92.1 cells. The original images of the blots in Figure 10a

## Data Availability

The datasets generated and analysed during the current study are available in the PRIDE repository, [ACCESSION NUMBER: PXD032215].

## Abbreviations

UM: Uveal melanoma
EGFP: Enhanced green fluorescent protein
TME: Tumor microenvironment
CQ: Chloroquine
DTIC: Dacarbazine
TMT: Tandem Mass Tags
SQSTM1: Sequestosome 1 (p62)
CM: Cutaneous melanoma

## References

[1] Rodriguezvidal C, Fernandezdiaz D, Fernandezmarta B (2020). Treatment of metastatic uveal melanoma: systematic review. Cancers.

[2] Louie BH, Kurzrock R (2020). BAP1: Not just a BRCA1-associated protein. Cancer Treat Rev.

[3] Aughton K, Kalirai H, Coupland SE (2020). MicroRNAs and uveal melanoma: understanding the diverse role of these small molecular regulators. Int J Mol Sci.

[4] Yang C, Wang Y, Hardy P (2020). Emerging roles of microRNAs and their implications in uveal melanoma. Cell Mol Life Sci.

[5] Baradaran PC, Kozovska Z, Furdova A (2020). Targeting Epigenetic Modifications in Uveal Melanoma[J]. Int J Mol Sci.

[6] Zhao DD, Zhao X, Li WT (2020). Identification of differentially expressed metastatic genes and their signatures to predict the overall survival of uveal melanoma patients by bioinformatics analysis. Int J Ophthalmol.

[7] Liu R, Chen Y, Liu G (2020). PI3K/AKT pathway as a key link modulates the multidrug resistance of cancers. Cell Death Dis.

[8] Choi S, Mihyang HA, Lee JS (2020). Novel prognostic factor for uveal melanoma: bioinformatics analysis of three independent cohorts. Anticancer Res.

[9] Wang F, Wang Q, Li N (2020). OSuvm: An interactive online consensus survival tool for uveal melanoma prognosis analysis. Mol Carcinog.

[10] Dong J, Zhang Q, Huang Q (2010). Glioma stem cells involved in tumor tissue remodeling in a xenograft model. J Neurosurg.

[11] Wang Z, Fei X, Dai X (2015). Differentiation of glioma stem cells and progenitor cells into local host cell-like cells: a study based on choroidcarcinoma differentiation of choroid plexus of GFP transgenic nude mouse. Cancer Biother Radiopharm.

[12] Fei XF, Qin ZH, Xiang B (2009). Olomoucine inhibits cathepsin L nuclear translocation, activates autophagy and attenuates toxicity of 6-hydroxydopamine. Brain Res.

[13] Lin L, Jia Z, Zhao Y (2017). Use of Limiting dilution method for isolation of nucleus pulposus mesenchymal stem/progenitor cells and effects of plating density on biological characteristics and plasticity[J]. Biomed Res Int.

[14] Fei X, Wang A, Wang D (2018). Establishment of malignantly transformed dendritic cell line su3-ihdctc induced by glioma stem cells and study on its sensitivity to resveratrol. BMC Immunol.

[15] Wan Y, Fei XF, Wang ZM (2012). Expression of miR-125b in the new, highly invasive glioma stem cell and progenitor cell line SU3. Chin J Cancer.

[16] Waard-Siebinga ID, Blom DR, Griffioen M (1995). Establishment and characterization of a uveal-melanoma cell line. International Journal of Cancer Journal International du Cancer..

[17] Lan Q, Chen Y, Dai C (2020). Novel enhanced GFP-positive congenic inbred strain establishment and application of tumor-bearing nude mouse model. Cancer Sci.

[18] Kanehisa M, Goto S (2000). KEGG: kyoto encyclopedia of genes and genomes. Nucleic Acids Res.

[19] Kanehisa M (2019). Toward understanding the origin and evolution of cellular organisms. Protein Sci.

[20] Kanehisa M, Furumichi M, Sato Y (2021). KEGG: integrating viruses and cellular organisms. Nucleic Acids Res.

[21] Tang Z, Li C, Kang B (2017). GEPIA: a web server for cancer and normal gene expression profiling and interactive analyses[J]. Nucleic Acids Res.

[22] Mariani P, Servois V, Pipernoneumann S (2012). Therapeutic options in metastatic uveal melanoma. Dev Ophthalmol.

[23] Nathan P, Cohen V, Coupland S, Curtis K, et al. United Kingdom Uveal Melanoma Guideline Development Working Group. Uveal Melanoma UK National Guidelines. Eur J Cancer. 2015 ;51(16):2404–2412. 10.1016/j.ejca.2015.07.013 26278648

[24] Steeb T, Hayani KM, Frster P (2020). Guidelines for uveal melanoma: a critical appraisal of systematically identified guidelines using the AGREE II and AGREE-REX instrument. Springer Open Choice.

[25] Heppt MV, Heinzerling L, Kähler KC (2017). Prognostic factors and outcomes in metastatic uveal melanoma treated with programmed cell death-1 or combined PD-1/cytotoxic T-lymphocyte antigen-4 inhibition. Eur J Cancer.

[26] Heppt MV, Amaral T, Kähler KC, et al. Combined immune checkpoint blockade for metastatic uveal melanoma: a retrospective, multi-center study. J Immunother Cancer. 2019; 7(1):299. 10.1186/s40425-019-0800-0 PMC6854774 31722735

[27] Pflugfelder A, Kochs C, Blum A, et al. Malignant melanoma S3-guideline “diagnosis, therapy and follow-up of melanoma.” J Dtsch Dermatol Ges. 2013;11:1–116. 10.1111/ddg.12113_suppl 24028775

[28] Swetter SM, Tsao H, Bichakjian CK (2019). Guidelines of care for the management of primary cutaneous melanoma. J Am Acad Dermatol.

[29] Pai M, Yeung C, Akl EA (2019). Strategies for eliciting and synthesizing evidence for guidelines in rare diseases. BMC Med Res Methodol.

[30] Theresa S, Anja W, Thomas R (2018). How to MEK the best of uveal melanoma: a systematic review on the efficacy and safety of MEK inhibitors in metastatic or unresectable uveal melanoma. Eur J Cancer.

[31] Truong A, Yoo JH, Scherzer MT (2020). Chloroquine sensitizes GNAQ/11-mutated melanoma to MEK1/2 Inhibition. Clin Cancer Res.

[32] Crabb JW, Hu B, Crabb JS (2015). iTRAQ quantitative proteomic comparison of metastatic and non-metastatic uveal melanoma tumors. Plos One.

[33] Zhang J, Yang Z, Dong J (2016). P62: an emerging oncotarget for osteolytic metastasis. J Bone Oncol.

[34] Wu Q, Xiang M, Wang K, et al. Overexpression of p62 induces autophagy and promotes proliferation, migration and invasion of nasopharyngeal carcinoma cells through promoting ERK signaling pathway. Current Cancer Drug Targets. 2020;20(8):624–637. 10.2174/1568009620666200424145122 32329689

[35] Jiang X, Huang Y, Liang X (2018). Metastatic prostate cancer-associated P62 inhibits autophagy flux and promotes epithelial to mesenchymal transition by sustaining the level of HDAC6. Prostate.

[36] Sample A, Zhao B, Qiang L (2017). Adaptor protein p62 promotes skin tumor growth and metastasis and is induced by UVA radiation. J Biol Chem.

[37] Karras P, Riveiro-Falkenbach E, Cañón E (2019). p62/SQSTM1 fuels melanoma progression by opposing mRNA decay of a selective set of pro-metastatic factors. Cancer Cell.

[38] Jager MJ, Shields CL, Cebulla CM (2020). Uveal melanoma. Nat Rev Dis Primers.

