# Supplementary material for: Proteomics analysis: inhibiting the expression of P62 protein by chloroquine combined with dacarbazine can reduce the malignant progression of uveal melanoma
Source: BMC Cancer. 2022 Apr 14;22:408. doi: 10.1186/s12885-022-09499-z (PMC9009011; doi:10.1186/s12885-022-09499-z)
Supplement: Supplementary file 1 — Additional file 1: Supplementary Figure 1. Expression of p62. The expression of p62 in 92.1-A cells was significantly higher than that in 92.1 cells. The original images of the blots in Figure 8. Supplementary Figure 2. Among the three plasmids with different targets, the expression of p62 decreased most obviously Interfered by si-P62#2. The original images of the blots in Figure 9a. Supplementary Figure 3. p62 expression was increased after p62 overexpression in 92.1 cells. The original images of the blots in Figure 10a [file 12885_2022_9499_MOESM1_ESM.docx]

**Supplementary Figures and Figure Legends**

**Supplementary Figure 1**

**
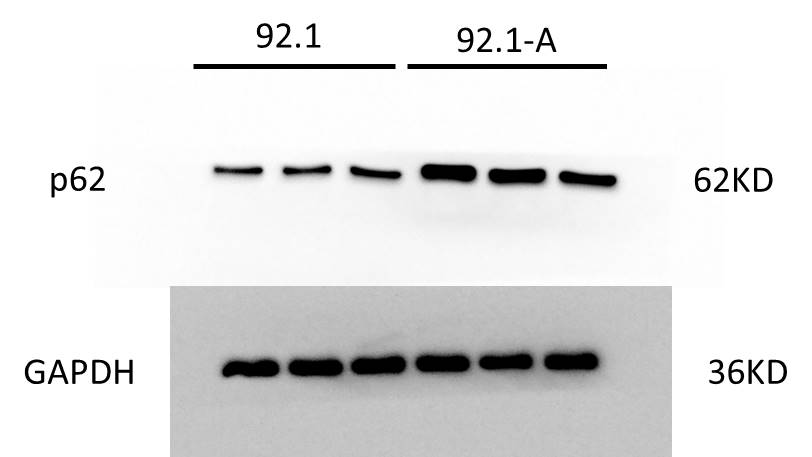
**

**Supplementary Figure 1,** **Expression of p62. The expression of p62 in 92.1-A cells was significantly higher than that in 92.1 cells**. The original images of the blots in Figure 8

**Supplementary Figure 2**

**
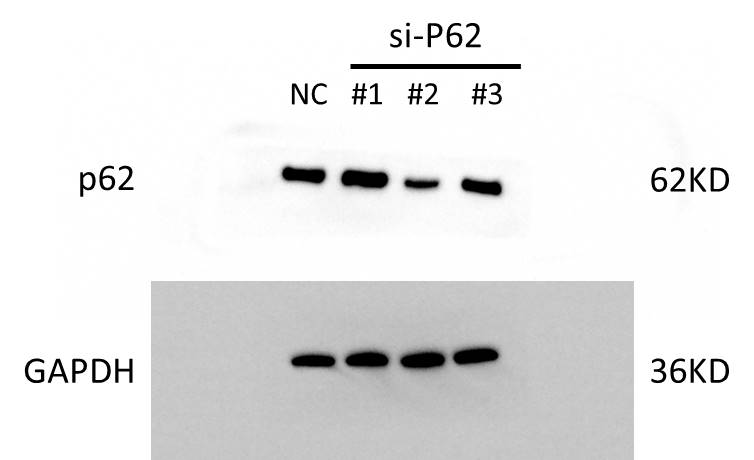
**

**Supplementary Figure 2, Among the three plasmids with different targets, the expression of p62 decreased most obviously Interfered by si-P62#2.** The original images of the blots in Figure 9a

**Supplementary Figure 3**


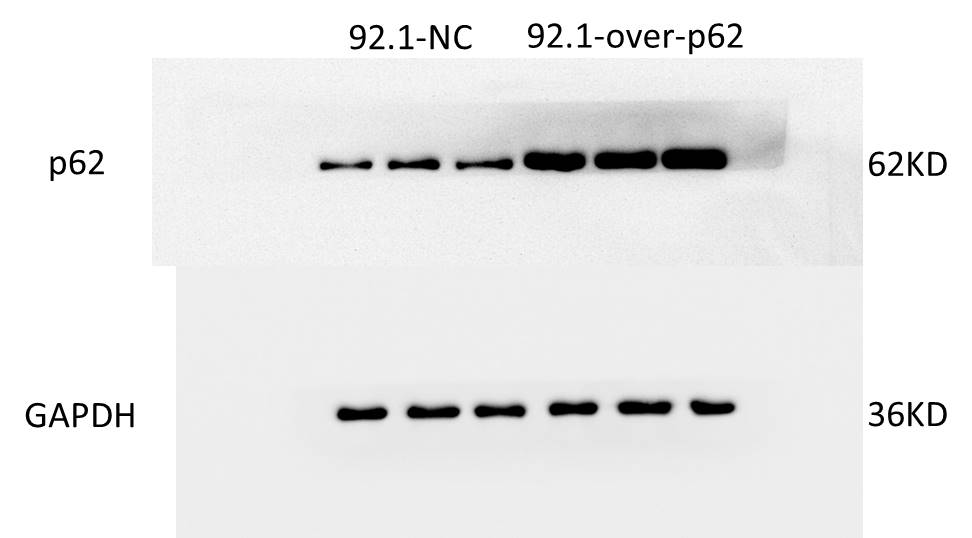


**Supplementary Figure 3,** p62 expression was increased after p62 overexpression in 92.1 cells. The original images of the blots in Figure 10a
